# High mobility group A2 is a target for miRNA-98 in head and neck squamous cell carcinoma

**DOI:** 10.1186/1476-4598-6-5

**Published:** 2007-01-14

**Authors:** Carla Hebert, Kathleen Norris, Mark A Scheper, Nikolaos Nikitakis, John J Sauk

**Affiliations:** 1 Diagnostic Sciences and Pathology, University of Maryland Baltimore, Baltimore Maryland 21201-1586, USA; 2 Diagnostic Sciences and Pathology, and Greenebaum Cancer Center, University of Maryland Baltimore, Baltimore Maryland 21201-1586, USA

## Abstract

**Background:**

HMGA2 expression has been shown to be associated with enhanced selective chemosensitivity towards the topoisomerase (topo) II inhibitor, doxorubicin, in cancer cells. Although the roles of signaling cascades and proteins as regulatory factors in development, neoplasia and adaptation to the environment are becoming well established, evidence for the involvement of regulatory small RNA molecules, such as microRNAs (miRNAs) as important regulators of both transcriptional and posttranscriptional gene silencing is presently mounting.

**Results:**

Here we report that HMGA2 expression in head and neck squamous cell carcinoma (HNSCC) cells is regulated in part by miRNA-98 (miR-98). Albeit HMGA2 is associated with enhanced selective chemosensitivity towards topoisomerase (topo) II inhibitor, doxorubicin in HNSCC, the expression of HMGA2 is thwarted by hypoxia. This is accompanied by enhanced expression of miRNA-98 and other miRNAs, which predictably target HMGA2. Moreover, we show that transfection of pre-miR-98™ during normoxia diminishes HMGA2 and potentiates resistance to doxorubicin and cisplatin. These findings implicate the role of a miRNA as a key element in modulating tumors in variable microenvironments.

**Conclusion:**

These studies validate the observation that HMGA2 plays a prominent role in governing genotoxic responses. However, this may only represent cells growing under normal oxygen tensions. The demonstration that miRNA profiles are altered during hypoxia and repress a genotoxic response indicates that changes in microenvironment in eukaryotes mimic those of lower species and plants, where, for example, abiotic stresses regulate the expression of thousands of genes in plants at both transcriptional and posttranscriptional levels through a number of miRNAs and other small regulatory RNAs.

## Background

High mobility group A2 (HMGA2) protein is a non-histone architectural transcription factor, which is a member of the HMGA family. This family is constituted by HMGA1a, HMGA1b, HMGA1c, and HMGA2[1,2]. HMGAs are chromosomal proteins that bind through their AT binding motifs or AT hooks to the minor groove of AT-rich DNA strands[3]. The two types of HMGA protein, HMGA1 (HMG-I/Y) and HMGA2 (HMGI-C), have similar functions and are coded for by two different genes at chromosomal loci 6p21.3 and 12q15, respectively. HMGA proteins are expressed predominantly during embryogenesis, their expression being absent or greatly reduced in adult tissues [4,5]. However, HMGA proteins have been implicated in the regulation of transcription, differentiation, neoplastic transformation, and integration and expression of viral genomes [6,7]. HMGA2 has been implicated mainly in benign and malignant mesenchymal tumors[8] Nevertheless, increased expression of HMGA2 in oral squamous cell carcinoma has been linked to epithelial-mesenchymal transition, whereby epithelial cells acquire mesenchymal characteristics [8]. Additionally, HMGA2 expression in patients with breast cancer has been shown to be associated with poor prognosis and metastasis [9]. However, the precise role of and the molecular events elicited by HMGA2 in tumorigenesis still need to be defined. Furthermore, HMGA2 expression has been shown to be associated with enhanced selective chemosensitivity towards the topoisomerase (topo) II inhibitor, doxorubicin, in breast cancer HS578T and salivary Pa-4/HMGA2 cells [2]. These studies have suggested that HMGA2 induces a persistent basal H2AX Ser139 phosphorylation and perturbs doxorubicin-elicited DNA damage checkpoint promoting enhanced chemosensitivity towards doxorubicin treatment in HMGA2-expressing cells.

Although proteins have been established as regulatory factors in growth, development and accommodation to alterations in environment, our knowledge of the contribution of regulatory small RNA molecules to such is rapidly evolving [10]. MicroRNAs (miRNAs) are 20–24 nucleotide non-coding RNA molecules that post-transcriptionally control gene expression. They are spawned by RNA polymerase II as large primary miRNA (pri-miRNA) transcripts [11,12]. Some miRNAs are structured in clusters and transcribed as polycistrons [13], while others exist in introns of protein-coding genes [14] and are co-transcribed with host genes. Pri-miRNA transcripts form typical fold-back structures that are identified and processed into ~70nt imperfect stem-loop miRNA precursors (pre-miRNAs) by a microprocessor complex [15,16]. After processing by the microprocessor complex, pre-miRNAs are exported by Ran-GTP dependent transporter exportin-5 [17-19] from the nucleus into cytoplasm, where they are processed by the RNAse III enzyme, Dicer, into imperfect dsRNA duplexes that contain both mature and complementary miRNA strands [20-22]. One of the mature miRNA strands forms an effector ribonucleoprotein complex termed RISC (RNA induced silencing complex) which guides the miRNAs to specific mRNAs [23]. RISC either cleaves or blocks translation of the target mRNA, depending on the degree of sequence complementarity.

Here we report that HMGA2 expression in head and neck squamous cell carcinoma (HNSCC) cells is regulated in part by the expression of miRNA-98 (miR-98). Albeit HMGA2 is associated with enhanced selective chemosensitivity towards topoisomerase (topo) II inhibitor, doxorubicin in HNSCC, the expression of HMGA2 is thwarted by hypoxia. This is accompanied by enhanced expression of miRNA-98 and other miRNAs, which predictably target HMGA2. Moreover, we show that transfection of pre-miR-98™ during normoxia diminishes HMGA2 and potentiates resistance to doxorubicin and cisplatin. These findings implicate the role of a miRNA as a key element in modulating tumors in variable microenvironments.

## Materials and methods

### Cell cultures

All experiments were performed using established cell lines, including primary human oral squamous cell carcinoma (SCC) cell lines from the American Type Culture Collection (SCC-4 and SCC-9), and the UMB-10B cell line (UMB). SCC cell lines were cultured in 1:1 mix of Ham's F12 and Dulbecco's Modified Eagle's Medium (DMEM) with 10% fetal bovine serum, 100 units of penicillin, 100 μg/ml streptomycin and 0.4 g/ml of hydrocortisone (Sigma Chemical Company). Cell lines were subcultured using a disaggregration assay with Trypsin (0.1%) and EDTA (0.01%) in phosphate buffered saline (PBS) pH 7.5. For some experiments cell lines were made hypoxic by placing them in a Heto-Holten Cell house incubator at 1% O_2_, 5% CO_2 _and 94% nitrogen for periods up to 1 hr or 5% O_2_, 5% CO_2 _and 90% nitrogen for periods up to 8 hr. All agents were administered by syringe in a closed system for hypoxia experiments to avoid reoxygenation effects. The oxygen levels for all systems were monitored using an Instech FO/SYS21OT micro oxygen monitoring system (Instech Labs., Inc.).

### Antibodies

Polyclonal monospecific mouse anti-HMGA2 antibody was obtained from ABNOVA. Monoclonal anti-actin antibody was from Chemicon. HRP-goat-antimouse secondary antibody was obtained from Kirkegaard & Perry Labs. Inc.

### RNA isolation, labeling and miRNA microarray

For these studies RNA was obtained using *mir*Vana™ miRNA Isolation Kit (Ambion). The levels and quality of RNA was achieved by the Agilent Bioanalyzer™ and with a spectrophotometer using 230 nm/260 nm ratios. The dendrimer Cy3 and Cy5 dyes were then employed to label RNA sequences. First, poly-A tails were added to the RNA sequences at 3' ends using poly(A) polymerase. A nucleotide tag was then added to the poly-A tail in a ligation reaction. In the case of dual-sample experiments, the two sets of RNA sequences were added with tags of two different sequences. The tagged RNA sequences were hybridized to the array with labeling carried out during the second hybridization reaction using dendrimer dyes. The labeled samples were then hybridized over night to a Human_V7.1C_051017 miRNA array chip (LC Sciences) using a micro circulation pump. After the hybridization, the chips were subjected to a stringent wash and fluorescence data were collected by using an Axon laser scanner model 4000B (Axon Instruments) and the chips were scanned at a pixel size of 10 μM with Cy3 Gain at 460 and the Cy5 Gain at 470 scanning. Data extraction and image process were performed using ArrayPro™ software equipped with a morphological filter (Media Cybernetics).

### Selection of plausible miR targets

To select plausible targets to validate the significance of the detected *miRs *we utilized 1) miRNA viewer [24], which is based on miRanda software for miRNA predication of potential targets (The default parameters were set at Gao Open Penalty:-8.0; Gap Extend: -2.0; Score Threshold: 50.0: Energry Threshold -20.0 kCal/mol; Scaling Parameter: 2.0) 2) *miRBase Targets *from the Sanger Institute, 3) TargetScanS, and 4) PicTar, which identifies both binding-sites targeted by single microRNA, as well as those that are co-regulated by several microRNAs in a coordinated manner. In that miRNAs may have a multitude of conserved miRNA:UTR pairs, recognizing that hypoxia has been associated with drug resistance, the general principle for these studies was to seek possible target genes that influenced DNA repair, drug action, or resistance.

### 3-(4,5-Dimethylthiazol-2-yl)-2,5-diphenyltetrazolium bromide assays for measurement of cell viability

For these studies, cell lines were seeded into 24-well plates to obtain a confluence of 50–60% on the day of the experiment. The cell lines were treated with various reagents of indicated concentration and medium was changed daily for 3 days. Twenty-four to seventy-two hours after the start of treatment (depending on the cell line), 50 μl of 5 mg/mL 3-(4,5-dimethylthiazol-2-yl)-2,5-diphenyltetrazolium bromide (MTT; Roche) in OptiMEM I (Invitrogen) was added to each well and the plate was incubated at 37°C for an additional 4 hours and 10% SDS in .01 M HCl was added to each well to dissolve the formazan crystals. Absorbance was immediately read at 575 nm in a scanning multiwell spectrophotometer. The results were depicted as percentage of cell viability, reported as the mean ± SD of three independent experiments done in triplicate.

### Protein lysate preparation and Western blot analysis

The cell lines were plated in 6-well plates using a density of 5 × 10^4^cells/well and were allowed to grow to 80% confluence. The cell lines were washed twice with cold PBS, lysed in RIPA buffer (50 mM Tris [pH 7.4], 150 mM NaCl, 1% Triton X-100, 1% deoxycholic acid, sodium salt, 0.1% sodium dodecyl sulfate [SDS], 100 μg/ml phenylmethylsulfonyl fluoride, 1 μg/ml aprotinin, 1 mM dithiothreitol and 1 mM sodium orthovanadate) for 10 min and scraped. The extracts were centrifuged at 40,000 *g *for 15 min at 4°C. Protein concentrations were measured and equalized using Bio-Rad protein assay (Bio-Rad Laboratories) according to the manufacturer's instructions. Equivalent amounts of protein (50 μg) were then separated by SDS-PAGE and then transferred to polyvinylidene difluoride membranes. Equivalent loading was confirmed by staining membranes with Ponceau-S. The membranes were blocked for 1 h in blocking buffer (1× Tris-buffered saline, 5% nonfat dry milk, and 0.1% Tween 20), which was subsequently replaced by the primary antibody in blocking buffer, overnight at 4°C. After incubation, the membranes were washed three times in washing buffer (1× Tris-buffered saline and 0.1% Tween 20). Primary antibody was detected using horseradish peroxidase-linked goat antimouse (Kirkegaard & Perry Labs. Inc.) or goat antirabbit IgG antibody (Kirkegaard & Perry Labs. Inc.) and visualized with SuperSignal West Pico chemiluminescent substrate. To validate loading of the gels the membranes were washed with PBS/Tween, treated with a stripping buffer (20 mMDTT, 2% SDS, 67.5 mM Tris, pH 6.7) rewashed with PBS/Tween, blocked with 10% non-fat dried milk, incubated with anti-β-actin antibodies and developed as described above. The bands were scanned and quantified using NIH image software.

### Transfection of small interfering RNA

For transient transfection, cell lines were seeded at 90% confluency, and transfections were carried out by using Lipofectamine 2000 (Invitrogen) according to the instructions of the manufacturer. The oligonucleotides encoding HMGA2 small interfering RNA (siRNA) were 5'-CAGCAATCTGTCGCTAAGGdTdT-3' and 5'-CCTTAGCGACAGATTGCTGdTdT-3'. In some studies SCC cell lines grown in D-MEM (GIBCO) supplemented with 10% fetal bovine serum (GIBCO) were transfected with 15 or 5 pmol of Pre-miR-98™ or negative control 1 Precursor miRNAs (Ambion) in 24-well plates using siPort Neo-FX™ (Ambion) according to the manufacturer's protocol. Three days posttransfection, cell lines were analyzed for viability and by Western blot. To study the effects of Anti-miR-98™, cell lines grown as above were transfected in 24-well plates with 30 pmol of Anti-miR-98™ or negative control 1 inhibitors (Ambion) using Lipofectamine 2000. Three days post transfection the cell lines were analyzed by vitality asays, by Northern blot and Western blot.

### Reverse transcription-PCR for HMGA2 and miRNA-98 and Northern blot validation of miRNA-98

RNA was extracted using Trizol reagent (Invitrogen) according to the protocols of the manufacturer followed by DNA-free DNase treatment (Ambion, Austin, TX). Subsequent cDNA synthesis and PCR reactions were carried out using ThermoScript reverse transcription-PCR (RT-PCR) System (Invitrogen). The following primer pairs were used for PCR reaction: HMGA2, 5'-GTGAGCCCTCTCCTAAGAGAC-3' and 5'-CTGCAGTGTCTTCTCCCTTC-3'; glyceraldehyde-3-phosphate dehydrogenase (GAPDH), 5'-GACCACAGTCCATGCCATCAC-3' and 5'-CATACCAGGAAATGAGCTTGAC-3'. To validate miRNA by PCR, RNA was extracted with the *mir*Vana™ miRNA Isolation Kit and the miRVana qRT-PCR miRNA™ Detection kit for miRNA-98 and 5S (Ambion). The levels of miRNA-98 were determined using mirVana™ Probe with oligonucleotide positive controls; 5S rRNA and an RNA probe specific for miR-16 miRNA following the manufactures protocols.

### Statistical analysis

Statistical analyses utilized two-tailed Student's test; statistical significance was set as *p *≤ 0.05.

## Results and discussion

**Figure 1:**
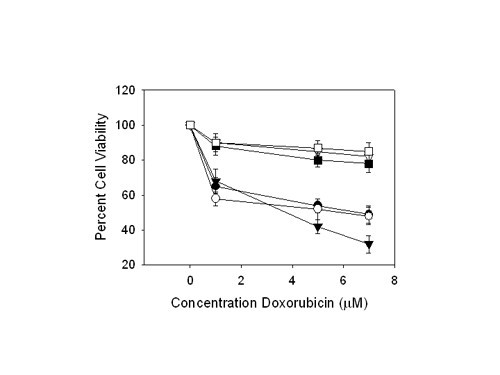
**Chemosensitivity towards doxorubicin of SCC cell lines**. Percent cell viability following doxorubicin treatment to assess chemosensitivity towards doxorubicin was measured by MTT assays. SCC cell lines were grown under normoxic (▽ SCC-4, ■ SCC-10B and □ SCC-9) or hypoxic (● SCC-4, ○ SCC-10B, and ▼ SCC-9) conditions and treated with various concentrations of doxorubicin. Significant differences between cell lines grown under hypoxic conditions and normoxic conditions was detected for all three cell lines as determined by two-tailed Student's *t *test (*p *≤ 0.05). The error bars represent the S.E.M. for 3 experiments.

Poor oxygenation (hypoxia) is present in the majority of human tumors and is associated with poor prognosis due to the protection it affords to radiotherapy and chemotherapy [25]. Here, we sought first to verify the chemoresistance effect of hypoxia to doxorubicin and cisplatin in SCC cell lines, comparing cell lines grown in normoxic and hypoxic atmospheres. These studies revealed all SCC cell lines studied here using MTT cell vitality responded in a dose-dependent manner to doxorubicin. Moreover, when the cell lines were grown under hypoxic conditions, they demonstrated enhanced cell viability (Figure 1). Similar results were also observed for cisplatin (not shown). The mechanisms for chemoresistance to these agents under hypoxia were initially unclear. Existing knowledge dictates that tumors cope with hypoxic microenvironments by bringing forth multiple cellular response pathways that alter gene expression and affect tumor progression by strongly suppressing the rates of mRNA translation during hypoxia [25].

Although the roles of signaling cascades and proteins as regulatory factors in development, neoplasia and adaptation to the environment are becoming well established, the evidence for involvement of regulatory small RNA molecules as important regulators of both transcriptional and posttranscriptional gene silencing is presently mounting [10]. Thus, in the present study we grew SCC cell lines both in a normoxic atmosphere and in an environment that mimics the hypoxia encountered in solid head and neck tumors and used differential display hybridization to a human miRNA chip, and Real-time RT-PCR and Northern blot validation, to discern miRNA expression profiles. SCC cells that were grown under hypoxic conditions differentially expressed 47 miRNA genes compared to cells grown under normoxic conditions (P < 0.04). The following miRNAs were expressed to the greatest extent during hypoxia:hsa-miRNA-572, -214, -563, -637, -628, -191, -210, -498, -373, -98, -148b, -15a, -148-a, -200a, -30b, -429, -7, hsa-let-7e, -7i, and -7g (Table 1). Conversely, the following miRNAs were most diminished in their expression in hypoxic cells: hsa-miRNA-122a, -565, -195, -30e-5p, -374, -19a, -101, -424, -29b, -186, -141, -320, -422b, and -197 (Table 1).

**Table 1:** miRNAs differentially expressed following hypoxia in SCC cells.

**miRNAs**	**Hypoxia Up-regulated**	**miRNAs**	**Hypoxia Down-regulated**
hsa-miRNA-572	6.16	hsa-miRNA-122a	-13.54
hsa-miRNA-214	5.39	hsa-miRNA-565	- 6.77
hsa-miRNA-563	517	hsa-miRNA-195	- 6.36
hsa-miRNA-637	4.96	hsa-miRNA-30e-5p	- 5.93
hsa-miRNA-98	4.56	hsa-miRNA-374	- 4.17
hsa-miRNA-628	4.50	hsa-miRNA-19a	- 3.95
hsa-miRNA-191	4.19	hsa-miRNA-101	- 3.73
hsa-miRNA-210	4.16	hsa-miRNA-424	- 3.68
hsa-miRNA-31	3.97	hsa-miRNA-186	- 3.63
hsa-miRNA-498	2.94	hsa-miRNA-29b	- 3.66
hsa-miRNA-373	2.85	hsa-miRNA-148b	- 3.48
hsa-miRNA-19a	2.46	hsa-miRNA-141	- 3.05
hsa-miRNA-148a	2.30	hsa-miRNA-22	- 2.10
hsa-miRNA-15a	2.28	hsa-miRNA-331	- 2.02
hsa-miRNA-200a	2.20	hsa-miRNA-422b	- 2.01
hsa-miRNA-7	2.20	hsa-miRNA-197	- 2.00
hsa-miRNA-30b	2.19		
hsa-let-7e	2.26		
hsa-let-7g	2.22		
hsa-let-7i	2.12		

SCC cells that were grown under hypoxic conditions differentially expressed 47 miRNA genes compared to cells grown under normoxic conditions (P < 0.04). Data represent the fold changes in miRNA expression. hsa-miRNA-98 represents the 5^th ^most highly expressed miRNA during hypoxia.

**Figure 2:**
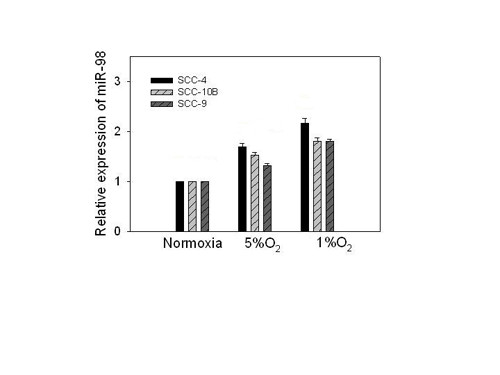
**Relative expression of miRNA-98 in normoxia and hypoxia**. The relative levels of miRNA-98 were determined using the *mir*Vana™ miRNA Isolation Kit and the miRVana qRT-PCR miRNA™ Detection kit for miR-98 and 5S (Ambion, Austin, TX). Cell lines were grown under normoxic or hypoxic (5% O_2 _and 1% O_2_) conditions. The differences between cell lines grown under normoxic condition were significantly different to those grown at 1% O_2 _with a *P *≤ 0.05. The error bars represent S.E.M. for 3 experiments.

One of the most prevalent changes in expression profiles involved a 4.56 fold increased expression of hsa-miR-98. To validate these findings we grew SCC cell lines in either a normoxic or hypoxic environment as before and assessed the miRNA-98 levels with miRVana qRT-PCR miRNA™ Detection kit. These studies revealed that following hypoxia the relative levels of miRNA-98 increased among the SCC cell lines in an oxygen dependent manner (Figure 2). These changes in miRNA-98 were validated in Northern blot analyses using mirVana miR-98™ Probe, normalized to 5S rRNA (not shown).

**Figure 3:**
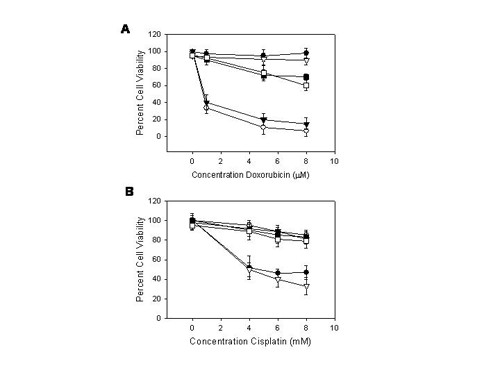
**a. Cell viability of doxorubicin treated SCC cell lines following pretreatment with anti-miR-98™ and pre-miR-98™**. The SCC-4 cell line was grown under normoxia or hypoxia and transfected with anti-miR-98™ or pre-miR-98™. The cell line was then challenged with varying doses of doxorubicin as described in *Methods*. The surviving fraction of cells as a percentage of control was determined using the MTT assay. Cell lines grown in normoxic conditions revealed greater percent of surviving cells when treated with pre-mir-98™ (●) compared to controls (▼) and anti-miR-98™ (○) transfected cells. (*p *≤ 0.05). There was no significant difference between cell lines treated with anti-miR-98™ and controls. Cell lines grown under hypoxic conditions and transfected with pre-miR-98™ (▽) showed a slight increase in surviving cells compared to control cell lines (■) and Anti-miR-98™ (□) transfected cell lines (*p *≤ 0.05). There was no significant difference between control and anti-miR-98™ transfected cell lines. Similar results were observed for SCC-9 and SCC-10B cell lines. The error bars represent S.E.M. for 3 experiments. **b. Cell viability of cisplantin treated cell lines following pretreatment with anti-miR-98™ and pre-miR-98™**. The SCC-4 cell line was grown under normoxic or hypoxic conditions and transfected with anti-miR-98™ while others were transfected with pre-miR-98™. The cell line was then challenged with varying doses of cisplatin as described in *Methods*. The surviving fraction of cells as a percentage of control was determined using the MTT assay. Cells grown in normoxic conditions revealed greater percent of surviving cells when treated with pre-mir-98™ (▼) compared to controls (●) and anti-miR-98™ (▽) transfected cell lines (*p *≤ 0.05). There was no significant difference between cells treated with anti-miR-98™ and controls. Cell lines grown under hypoxic conditions and transfected with pre-miR-98™ (■) showed no significant difference in the percent of surviving cells compared to control cells (○) and Anti-miR-98™ (□) transfected cell lines. Likewise, there was no significant difference between control and anti-miR-98™ transfected cell lines. Similar results were observed for SCC-9 and SCC-10B cell lines. The error bars represents S.E.M. for 3 experiments.

To further appraise the role of miRNA-98 during normoxia and hypoxia SCC cell lines were transfected with the pre-miR-98™ or anti-miR-98™ (Ambion) using siPORT™ NeoFX™ Transfection Agent, for 72 hours. The predicted changes in the levels of miRNA-98 were validated by miRVana qRT-PCR™ as before (not shown). SCC cell lines transfected with either anti-miR-98™ or with pre-miR-98™ were then challenged with varying doses of doxorubicin and the surviving fraction of cells as a percentage of control was determined using the MTT assay. Cell lines grown in normoxic conditions revealed greater percent of surviving cells when treated with pre-miR-98™ compared to controls and anti-miR-98™ transfected cell lines. There was no significant difference between cell lines treated with anti-miR-98™ and controls. Cell lines grown under hypoxic conditions and transfected with pre-miR-98™ showed a slight increase in surviving cells compared to control cells and anti-miR-98™ transfected cell lines. There was no significant difference between control and anti-miR-98™ transfected cell lines (Figure 3a). Similar findings were also extended to other double-strand breaks elicited by cisplatin in SCC cells (Figure 3b). These studies revealed that normoxic cell lines treated with pre-miR-98™ acquired chemoresistance to doxorubicin and cisplatin.

**Figure 4:**
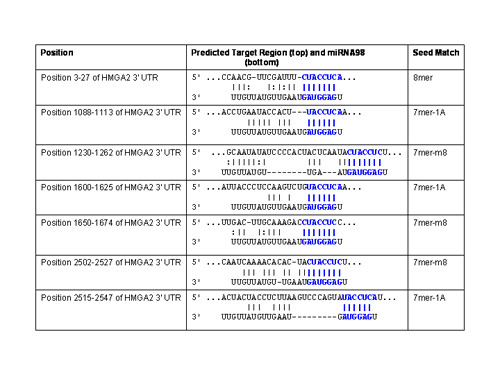
**TargetScanS miRNA98 target sites for HMGA2**. In addition to 7mer sites, TargetScanS predicts 8mer sites defined as: An exact match to positions 2–8 of the mature miRNA (the seed + position 8) with a downstream 'A' across from position 1 of the miRNA. In addition to 8mer sites, TargetScanS predicts 7mer sites of two types: 7mer-m8: An exact match to positions 2–8 of the mature miRNA (the seed + position 8) and 7mer-1A: An exact match to positions 2–7 of the mature miRNA (the seed) with a downstream 'A' across from position 1 of the miRNA.

**Figure 5:**
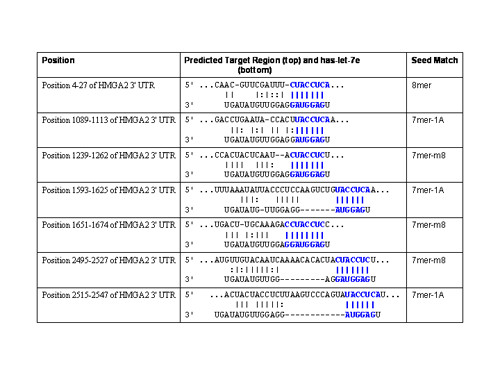
**TargetScanS has-let-7g target sites for HMGA2**. In addition to 7mer sites, TargetScanS predicts 8mer sites defined as: An exact match to positions 2–8 of the mature miRNA (the seed + position 8) with a downstream 'A' across from position 1 of the miRNA. In addition to 8mer sites, TargetScanS predicts 7mer sites of two types: 7mer-m8: An exact match to positions 2–8 of the mature miRNA (the seed + position 8) and 7mer-1A: An exact match to positions 2–7 of the mature miRNA (the seed) with a downstream 'A' across from position 1 of the miRNA.

**Figure 6:**
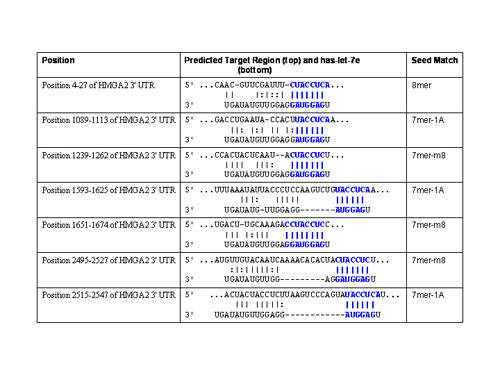
**TargetScanS has-let-7e target sites for HMGA2**. In addition to 7mer sites, TargetScanS predicts 8mer sites defined as: An exact match to positions 2–8 of the mature miRNA (the seed + position 8) with a downstream 'A' across from position 1 of the miRNA. In addition to 8mer sites, TargetScanS predicts 7mer sites of two types: 7mer-m8: An exact match to positions 2–8 of the mature miRNA (the seed + position 8) and 7mer-1A: An exact match to positions 2–7 of the mature miRNA (the seed) with a downstream 'A' across from position 1 of the miRNA.

**Figure 7:**
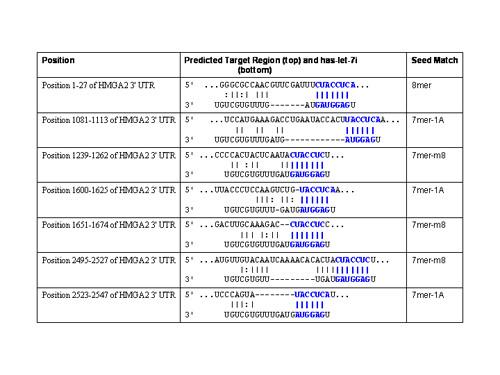
**TargetScanS has-let-7i target sites for HMGA2**. In addition to 7mer sites, TargetScanS predicts 8mer sites defined as: An exact match to positions 2–8 of the mature miRNA (the seed + position 8) with a downstream 'A' across from position 1 of the miRNA. In addition to 8mer sites, TargetScanS predicts 7mer sites of two types: 7mer-m8: An exact match to positions 2–8 of the mature miRNA (the seed + position 8) and 7mer-1A: An exact match to positions 2–7 of the mature miRNA (the seed) with a downstream 'A' across from position 1 of the miRNA.

Next, we sought to assess potential targets for miRNA-98 that might explain the observed chemoresistance to these drugs during hypoxia or in pre-miR-98™ transfected normoxic cell lines. In that miRNA-98 has 487 conserved miRNA:UTR pairs within the human genome by *miRNA viewer*, we selected potential targets that were predicted from at least two of the database resources with at least two miRNA target sites. Utilizing *miRNA viewer*, the high mobility group protein HMGI-C (High mobility group AT-hook 2, HMGA2) was one of the highest ranking genes with five potential target sites for *hsa-miRNA-98*. Utilizing other databases it was found that *PicTar *defined 6 *hsa-miRNA-98 *target sites, and *TargetScanS *identified 7 *hsa-miRNA-98 *target sites (Figure 4). Notable was that a number of other miRNAs included in the hypoxia profile also were potential regulators for HMGA2. Most prevalent among this later group utilizing *TargetScanS *were hsa-let-7g which possessed 7 target sites for HMGA2 (Figure 5), hsa-let-7e with 7 target sites (Figure 6) and hsa-let-7i with 7 sites (Figure 7). Albeit, the expression of has-let-7g, -7i, and -7i was similar to miRNA-98, being low in normoxia, but increasing following hypoxia (Table 1).

**Figure 8:**
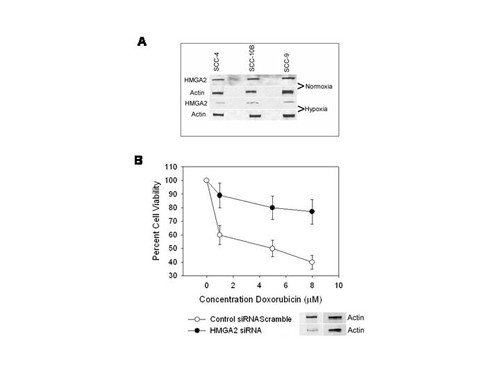
**a. Effect of hypoxia and normoxia on the protein expression of HMGA2 by Western blot**. SCC-4, SCC-9 and SCC-10B cell lines were grown under normoxic and hypoxic conditions (1% O_2_), harvested and the levels of HMGA2 assayed by Western blot, as described in *Methods*. **b. Effect of HMGA2 small interfering RNA on cell viability following doxorubicin treatment in SCC cell lines**. The SCC-4 cell line grown under normoxic conditions was transfected with oligonucleotide encoding HMGA2 small interfering RNA or a scramble siRNA sequence. The dose dependent effects of doxorubicin on cell viability was measured by MTT assays. The differences between control cell line and cell lines treated with HMGA2 siRNA was statistically significant (*p *≤ 0.01) as determined by Student's *t *test. The panel below depicts the effects of HMGA2 siRNA and the scramble counterpart on HMGA2 in Western blot. Similar results were obtained for SCC-9 and SCC-10B cell lines. The error bars represent the S.E.M. for 3 experiments.

Recognizing that HMGA2 has been shown to perturb doxorubicin-elicited DNA damage checkpoint control and to promote enhanced chemosensitivity towards doxorubicin and cisplatin treatment in HMGA2-expressing breast cancer cells [2], we assessed whether hypoxia altered the expression of HMGA2; SCC cell lines were subjected to hypoxia, harvested and the expression of HMGA2 was determined by Western blot. These studies revealed that hypoxia diminished the levels of HMGA2 protein expression (Figure 8a).

**Figure 9:**
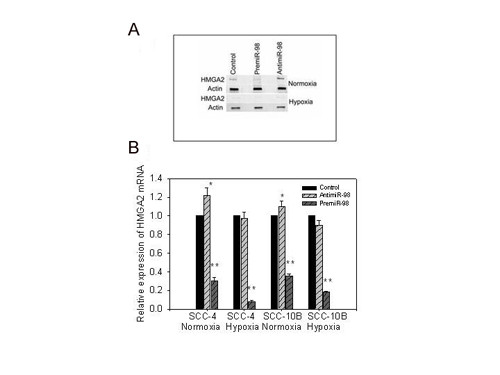
**a. The effect of pre-miR-98™ and anti-miR-98™ on the protein levels of HMGA2 during normoxia and hypoxia**. The SCC-4 cell line was transfected with pre-miR-98™ or Anti-miR-98™ and grown under hypoxic or normoxic conditions. Similar results were obtained for SCC-10B and SCC-9 cell lines (not shown). **b. The effect of pre-miR-98™ and anti-mir-98™ on the relative expression of HMGA2 during normoxia and hypoxia**. The mRNA expression of HMGA2 was significantly diminished both during normoxia and hypoxia following transfection with pre-miR-98™ (** *p *≤ 0.01). Anti-miR98™ transfection had a significant effect on enhancing HMGA2 expression when cells lines were grown during normoxia (*p ≤ 0.05) but were ineffective under hypoxic conditions. The mRNA expression of the control levels of each condition was set as 1. The error bars represents S.E.M. for 3 experiments.

To verify that HMGA2 potentiated genotoxic stress in SCC cell lines we paralleled our studies to those performed in breast cancer cells [2]. In essence, cell lines were transfected with siRNA oligonucleotides for HGMA2 as described above. The oligonucleotides encoding HMGA2 siRNA was compared with an oligonucleotide scramble siRNA as control [2]. These studies revealed that HMGA2 siRNA induced down-regulation of HMGA2 protein expression as assessed by Western blot (Figure 8b lower panel), and diminished doxorubicin sensitivity by MTT assays (Figure 8c). Recognizing that SCC cell lines became resistant to doxorubicin during periods of hypoxia and following diminished HMGA2 expression after siRNA transfection in normoxic cells we sought to verify that HMGA2 was a feasible target for miRNA-98 in SCC cell lines. To further appraise HMGA2 as a miRNA-98 target, SCC cell lines were transfected with the pre-miR-98™ (Ambion) using siPORT™ NeoFX™ Transfection Agent, for 72 hours. The increased levels of miRNA-98 were validated by miRVana qRT-PCR™ as before. These treatments resulted in diminishment in protein levels and mRNA expression of HMGA2 in control the normoxic SCC-4 cell line as well as cell lines exposed to hypoxia (Figure 9a,b); similar results were obtained for SCC-10B and SCC-9 cell lines (not shown). The demonstration that pre-miRNA-98™ diminished mRNA expression of HMGA2 was surprising, since the tenet of early studies, using reporter constructs had indicated that complete complementarities between miRNAs and target mRNAs was necessary for cleavage, whereas mismatches result in translational repression. However, the findings reported here are supported by more recent research, which revealed that miRNAs with partial complementarities can induce attenuation in cognate transcript levels [26-29]. In fact, most recently it has been shown that the ability of miRNAs to expedite poly(A) removal does not result from decreased translation; nor does translational repression by miRNAs require a poly(A) tail, with a 3' histone stem-loop being an effective substitute [30].

Conversely, the use of the anti-miR-98™ provided little information in the present studies. Cell lines grown under normoxia express little miRNA-98 so that the effects of anti-mir-98™ on HMGA2 levels were limited (Figure 9a,b). Transfection with anti-miR-98™ had only minor effects on the HMGA2 levels in SCC cell lines grown in hypoxia (Figure 9a,b). This is probably owing to the increased expression during hypoxia of other targeting miRNAs such as *hsa – let-7g*, *hsa-let-7e *and *hsa-let-7i *all of which possess 7 complementary target sites for HMGA2. Inherent in these findings is the recognition that derepression of a mRNA targeted by a miRNAs in an altered microenvironment probably can not be achieved through down-regulation of a single miRNA in that most mRNAs possess multiple complementary target sites with a number of miRNAs.

Until lately, there was only speculation that inhibition by a miRNA to a specific mRNA could be effectively reversed. However, recent studies have shown that CAT-1 mRNA and reporters bearing its 3'UTR can be relieved from miR-122-mediated repression in human Huh7 hepatoma cells when they are subjected to different types of stress. Moreover, that the derepression is accompanied by the release of CAT-1 mRNA from cytoplasmic processing bodies and its entry into polysomes and that the process involves binding of HuR, *trans*-acting AU rich element (ARE) binding protein, to the 3'UTR of CAT-1 mRNA [31]. There is also additional evidence indicating that some miRNA-controlled mRNAs can be relieved from repression by synaptic stimulation [32,33] indicating that miRNA regulation is more pronounced than previously anticipated and that it is able to respond quickly to specific cellular needs. What is yet to be determined is whether changes in oxygen tension provide a like mechanism for derepression of miRNAs against HMGA2. There is clear evidence that HuR, a major ARE-binding protein is expressed in many different cells and regulates the stability, subcellular shuttling, and/or translation of ARE-mRNAs (reviewed [34]). Moreover, the level of cytoplasmic HuR increases during cell proliferation, differentiation, and in response to stress stimuli [35-38] decreasing with senescence [39]. Also, the high level of cytoplasmic HuR in cancer cells is in keeping with the ability of HuR to increase the stability and translation of ARE-mRNAs that regulate cell proliferation and neovascularization by stabilizing VEGF following hypoxia. HuR has also been shown to shuttle between nucleus and cytoplasm during hypoxia and to stabilize HIF-1α and EGFR mRNAs [34]. Consequently, we searched the AERD database [40] but were not able to demonstrate any of the five clusters containing adenylate uridiylate (AU)-rich elements in their 3'-untranslated region (3'-UTR) that would afford HMGA2 stabilization or derepression by HuR. Furthermore, since HuR shuttles from the nucleus to the cytoplasm during hypoxia it would appear that HMGA2 would be overwhelmed by HuR, making it unlikely that HMGA mRNA is stabilized like other ARE-mRNAs.

## Conclusion

These studies validate the observation that HMGA2 plays a prominent role in governing genotoxic responses. However, this may only represent cells growing under normal oxygen tensions. The demonstration that miRNA profiles are altered during hypoxia and repress a genotoxic response indicates that changes in microenvironment in eukaryotes mimic lower species and plants, where, for example, abiotic stresses regulate the expression of thousands of genes in plants at both transcriptional and posttranscriptional levels through a number of miRNAs and other small regulatory RNAs[41]. Yet to be established is to what extent miRNA expression is regulated by defined signaling cascades and transcription factors.

## Authors' contributions

C H performed Western blots, Gene transfection

KN maintained Cell Cultures and performed Drug assays

MS performed miRNA target analysis

NN carried out data analysis and was co-writer

JJ initiated research design performed data analysis and was a co-writer
